# Survey of aphid population in a yellow passion fruit crop and its relationship on the spread *Cowpea aphid*-*borne mosaic virus* in a subtropical region of Brazil

**DOI:** 10.1186/s40064-015-1263-5

**Published:** 2015-09-22

**Authors:** Renata Maia Garcêz, Alexandre Levi Rodrigues Chaves, Marcelo Eiras, Laura Maria Molina Meletti, Joaquim Adelino de Azevedo Filho, Leonardo Assis da Silva, Addolorata Colariccio

**Affiliations:** Laboratório de Fitovirologia e Fisiopatologia (LFF), Instituto Biológico (IB), São Paulo, SP Brazil; Instituto Agronômico de Campinas (IAC), Centro de Pesquisa e Desenvolvimento de Recursos Genéticos Vegetais (CPDRGV), Campinas, SP Brazil; Agência Paulista de Tecnologia dos Agronegócios (APTA), Polo Leste Paulista, Monte Alegre do Sul, SP Brazil

**Keywords:** Monitoring, Aphids, Vector-borne, CABMV, *Potyvirus*, Passifloraceae

## Abstract

**Background:**

Passion fruit woodiness may be caused by *Cowpea aphid*-*borne mosaic virus* (CABMV) and is currently the major passion fruit disease in Brazil. To assess the virus-vector-host interactions, a newly introduced golden passion fruit plantation located in eastern region of São Paulo State, Brazil, was monitored.

**Methods:**

Dissemination of CABMV was determined analyzing golden passion fruit plants monthly for 18 months by PTA-ELISA. Seasonality and aphid fauna diversity was determined by identification of the captured species using yellow sticky, yellow water-pan and green tile traps. Population composition of the aphid species was determined using the descriptive index of occurrence, dominance and general classification and overlap of species in the R program.

**Results:**

Analyses of species grouping afforded to recognize 14 aphid species. The genus *Aphis* represented 55.42 % of the species captured. Aphid species formed two distinct clusters, one of which was characterized by the diversity of polyphagous species that presented high potential to spread CABMV.

**Conclusion:**

The low abundance and diversity of aphid species did not interfere negatively in the CABMV epidemiology. The genus *Aphis*, particularly *Aphis fabae/solanella* and *A. gossypii*, was crucial in the spread of CABMV in passion fruit orchards in the eastern State of São Paulo.

## Background

Today, tropical fruit cultures are among the most important agricultural activities in Brazil. The increasing demand for fresh fruit by high street markets and especially by the juice industry has boosted the development of new technologies that favour the expansion of passion fruit (*Passiflora edulis* Sims) plantations. Currently, Brazil holds the first place in passion fruit production worldwide, with 920,000 tons produced per year in an area of approximately 62,019 hectares (AGRIANUAL 2013).

The substantial intensification of passion fruit culture has exposed plants to several diseases, most of which are caused by viruses. Specifically in Brazil, several viruses have been reported to infect passion fruit, such as the *Cucumber mosaic virus* (CMV, *Cucumovirus*), *Passion fruit veinclearing virus* (PVCV, *Potyvirus*), *Purple granadilla mosaic virus* (PGMV, not yet taxonomically ranked), *Passion fruit green spot virus* (PFGSV, *Rhabdovirus*), *Passion fruit yellow mosaic virus* (PFYMV, *Tymovirus*), *Cowpea aphid*-*borne mosaic virus* (CABMV, *Potyvirus*) and Passion fruit severe leaf distortion virus (PFSLDV, *Begomovirus*) (Chagas et al. 1981; Nascimento et al. 2006; Fischer and Rezende 2008; Ferreira et al. 2010).

In the past, CABMV isolates in Brazil were identified as *Passionwoodiness virus* (PWV, *Potyvirus*) (Chagas et al. 1981). Originally, PWV was described in Australia as the only virus able to induce hardening of fruits (Taylor and Greber 1973). Later, however, Brand et al. (1993) sequenced the coat protein gene of a PWV isolate from South Africa and compared it with an isolate from Australia, concluding that both isolates were CABMV. Since then, comparative studies on nucleotide sequencing of coat protein revealed that Brazilian isolates, formerly described as PWV, had high identity with CABMV (Nascimento et al. 2006; Rodrigues et al. 2015).

In the field, CABMV, like potyviruses, is efficiently disseminated by aphid vectors in a non-circulative, non-persistent manner, using the “helper strategy” (Ng and Falk 2006; Brault et al. 2010; Bragard et al. 2013). Approximately 75 % of aphid-vectored viruses are transmitted in a non-persistent manner (Powell 2005). The aphid species *Myzus persicae* (Sulzer), *Aphis gossypii* (Glover), *Aphis fabae*/*solanella* (Scopoli), *Aphis craccivora* (Bock), *Toxoptera citricidus* (Kilkaldy), *Uroleucon ambrosiae* (Thomas) and *Myzus nicotianae* (Blackman) are known CABMV vectors in passion fruit (Costa et al. 1995; Inoue et al. 1995), while *Ropalosiphum maidis* (Fitch), *A. gossypii*, *Macrosiphum euphorbiae* (Thomas), *M. persicae* and *Acyrtosiphon pisum* (Harris), are vectors of CABMV in cowpeas (Bashir et al. 2002).

Currently, annual renewal of crops is recommended in an effort to reduce losses due to the high incidence of CABMV in Brazilian passion fruit crops. However, other measures are also adopted, such as the cultivation of plants in anti-aphid glasshouses, the use of plants of at least 80 cm in height to establish plantations, and the establishment of new crops in areas where passion fruit has never been grown, in an effort to delay the introduction of the virus (Meletti 2011). Management of CABMV in crops takes into consideration a series of factors, like the vector-host interaction, the disease status, and environmental variables prevailing in a given region (Inoue et al. 1995, Barros et al. 2011). In this sense, strategies to monitor the arthropod fauna, temperature, rainfall, virus incidence in crops and spontaneous host plants have been developed. Although studies on diseases affecting passion fruit are scarce, it is well known that climate variables play an important role in the biological cycle of aphid species, limiting or increasing population density and governing assemblage formation and settlement patterns (Robert 1987). The abundance and occurrence of aphid species in different seasons of the year may also be associated with food resources supplies, and any environmental disturbance that affect these factors will also have an effect on population density and behavior of different aphid species in an environment (Wolda 1978).

This study evaluates the population dynamics of aphid species (diversity, population grouping and temporal distribution) using different traps, and monitors CABMV incidence in a passion fruit plantation in eastern region of São Paulo State (SP), Brazil, considering climate data, in an attempt to contribute for CABMV management.

## Methods

### Field studies: experimental layout, environmental conditions and assessment of aphid population and CABMV incidence

Passion fruit seedlings were obtained from certified virus-free seeds of hybrid IAC-275 kept in the Germoplasm Bank, Agronomical Institute of Campinas (IAC). Seedlings were grown in a greenhouse equipped with an anti-aphid screen. Before transplanting, 60 days after sowing, seedlings were indexed by *Plate Trapped Assay*-*Enzyme Linked Immunosorbent Assay* (PTA-ELISA), using specific polyclonal antisera against CABMV (Nascimento et al. 2006). Absorbance was read at 405 nm in an ELISA reader (Microplate Reader 3550-UV, Bio-Rad) in triplicates, after the application of p-nitrophenylphosphate as substrates. Results were expressed as the ratio of mean absorbance of samples infected to mean absorbance of healthy samples (negative controls). Samples were considered positive when mean absorbance readings were at least three times as high as negative control absorbance values. To comply with technical recommendations established by local regulations, seedlings were transplanted after reaching about 80 cm in height (Meletti 2011).

To determine the presence of CABMV in passion fruit orchards, single point disease assessments were performed (Nutter 1997). This evaluation comprised a survey to identify CABMV, its occurrence, concentration, prevalence, incidence and severity, which afforded an overview of this pathogen’s status in eastern SP, the region where it was recently introduced. Samples were collected in passion fruit orchards in the municipalities of Monte Alegre do Sul (22°40′55″S; 46°40′51″W, 750 m a.s.l.) and Pinhalzinho (22°46′46″S; 46°35′26″W, 910 m a.s.l.), SP, Brazil.

**Fig. 1:**
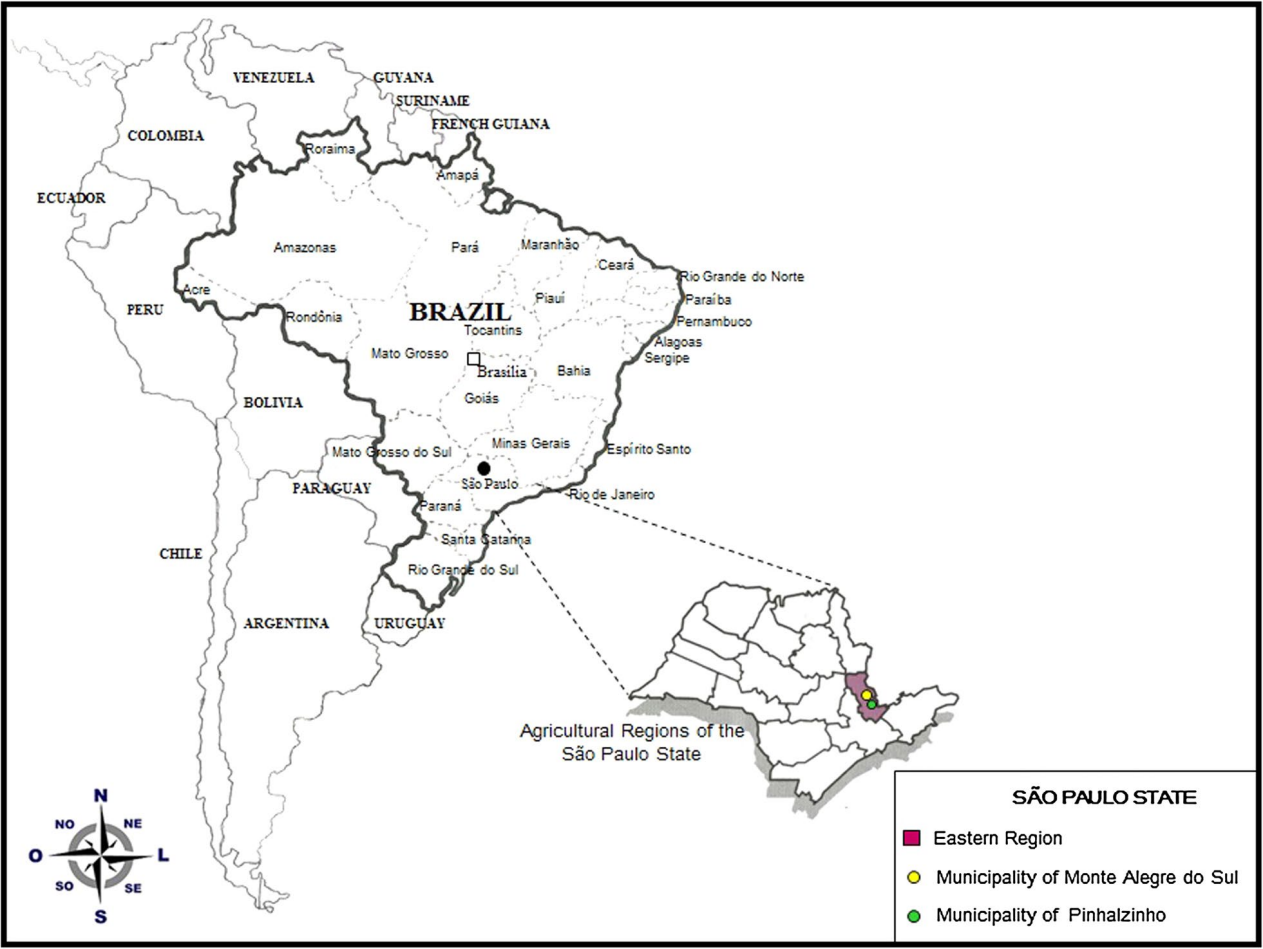
Map of Brazil, showing São Paulo State (*inset*) and the municipalities of Monte Alegre do Sul and Pinhalzinho, where the aphid fauna and the incidence of the *Cowpea aphid*-*borne mosaic virus* (CABMV) were surveyed

A field survey on CABMV and on aphid population dynamics was carried out in 100 fixed passion fruit plants grown in a 1000-m^2^ area in a passion fruit farm in the municipality of Pinhalzinho (Fig. 1). Surveys lasted 18 months (May 2010 to October 2011). To assess the influence of environmental variables in the monitored orchard, mean temperature (°C) and rainfall (mm) for Pinhalzinho were obtained daily accessing the Brazilian Space Agency webpage (http://www.inpe.br) daily during the study period.

The quantitative distribution of CABMV in the area was evaluated using the multiple point disease assessment described by Nutter (1997). The incidence of the target pathogens (ID-CABMV) was determined monthly by dividing the number of infected passion fruit plants by the total number of evaluated plants ×100, according to the methodology proposed by Gibbs (1983). Up to the fourth month, CABMV occurrence was assessed using individual fragments of the apical leaf. After, passion fruit plants were allowed to grow to a sole stem. From the fifth month on, the stem was induced to bifurcate, so as to form trellises and then bear fruit. Then, four fragments (two leaves for each bifurcated stem) per plant were collected. PTA-ELISA was used to detect CABMV in the passion fruit orchard.

Aphids flying over the monitored area were captured to investigate diversity, population dynamics and temporal for estimation of species richness. For that end, two yellow sticky traps were used to catch the total of specimens, and two yellow water-pan traps (Moericke 1955) were placed on the north and south sides of the field. Moreover, in order to determine potential vectors species, a green tile trap (Irwin 1999) was placed in the centre of the monitored area. The number of traps of each kind used was small, since it does not significantly affect the number of species and individuals collected (Ilharco 1992).

### Aphid species identification and descriptive indices used in diversity analysis and classification of aphid fauna

Stereomicroscope and dichotomic identification keys for recurrent aphids in the monitored area were used to identify aphid species and establish accurate numbers or estimates of aphids caught in traps (Eastop et al. 1993; Costa et al. 1993; Blackman and Eastop 2000; Claude and Guy 2002). The identification and preservation of the captured aphids were carried out according to Ilharco and Gomes (1981).

Based on species diversity and its temporal and spatial distribution, species richness and evenness estimates were obtained by cumulative catches of aphids (m^2^) using the Shannon’s index (H′) (Shannon 1948) according to the formula H′ = −Σp_i_ log_2_ p_i_, where “p_i_” corresponds to the number of target species (n_i_) on total number of individuals captured (N). The scale to express the diversity values is: H′ > 3.0 (high diversity); 3.0 < H′ > 2.0 (average diversity); 2.0 < H′ > 1.0 (low diversity) and H′ < 1.0 (very low diversity). The indices ‘Occurrence’ and ‘Dominance’ were employed to describe aphid species captured using yellow water-pan traps and green tile traps (Abreu and Nogueira 1989). Occurrence was calculated dividing the number of samples where one aphid species was recorded by the total number of samples, multiplied by 100. This allowed grouping aphid species into accidental (0–25 %), accessory (26–50 %) and constant (51–100 %). Dominance index was estimated dividing the number of individuals of one species by the total number of individuals, multiplied by 100. Using the data collected, aphid species were grouped into accidental (0–2.5 %), accessory (2.6–5.0 %) and dominant (5.1–10.0 %). The combination of occurrence indices and dominance allowed determining the general classification (or status) of the captured aphid species, which were classified into ‘Common’ (Constant + Dominant), ‘Intermediate’ (Constant + Accessory; Constant + Accidental; Accessory + Accidental; Accessory + Dominant; Accessory + Accessory), and ‘Rare’ (Accidental + Accidental). The clustering of aphid species in the study area was obtained using the Jaccard similarity index (available in the software R).

### Transmission tests

Aphid-borne virus transmission was assessed using the species *A. fabae*/*solanella* and *Uroleucon sonchi* (Linné). Colonies thereof were obtained from *Solanum americanum* (Mill.) “blackberry nightshade” (Solanaceae) and *Sonchus oleraceus* (L.) “common sowthistle” (Asteraceae), respectively, which are weeds commonly colonized by these aphids species in the monitored area. Inocula were obtained using passion fruit IAC-275 mechanically inoculated with the CABMV from the surveyed area. The aphids obtained in these sampling efforts were maintained under controlled conditions in their host plants, grown from seeds in a greenhouse. Transmission assays were carried out as three treatments with 10 seedlings of passion fruit each, with five repeats, allowing 5 min for virus acquisition and 10 min for transmission. Aphids not submitted to the CABMV acquisition period were used as negative controls. For each passion fruit plant, 10 apterous adult aphids, previously submitted to a 1-h pre-acquisition fasting period were used. After the transmission step, these aphids were manually removed and seedlings were kept in a greenhouse to investigate transmission efficiency. Viral infection signs in passion fruit seedlings thus challenged were assessed, and seedlings were submitted to PTA-ELISA to confirm CABMV infection.

## Results and discussion

### Multiple point and individual point disease assessment

The multiple point disease assessment carried out in a passion fruit plantation in eastern São Paulo State revealed that CABMV occurs in the region. However, the results also reveal that CMV and other aphid-borne viruses do not occur (data not shown) in the orchards analyzed, indicating that CABMV is the predominant virus infecting passion fruit plantations in the region.

**Fig. 2:**
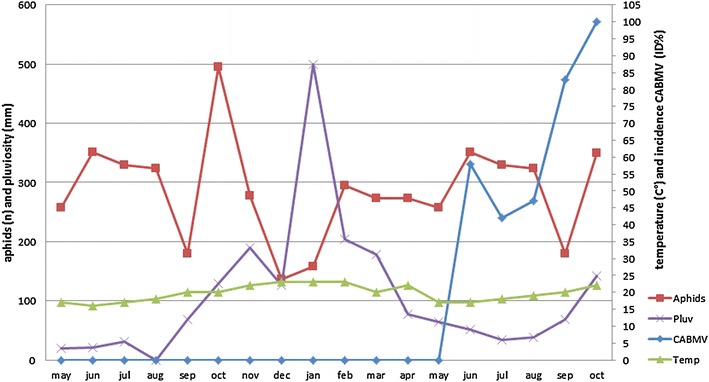
Monthly mean temperatures and rainfall values and winged aphid species recorded using sticky yellow traps and incidence of (ID-CABMV %) the *Cowpea aphid*-*borne mosaic virus* (CABMV) in a passion fruit plantation in Pinhalzinho, eastern São Paulo State, Brazil determined by PTA-ELISA

The individual point disease assessment of individual samples collected from the passion fruit plants monitored in Pinhalzinho, eastern SP by PTA-ELISA showed that, from the 14th month on, 57 % of the asymptomatic plants assessed were infected with CABMV. From the 15th month on, typical CABMV infection signs like foliar mosaic were observed in between 42 and 46 % of the plants examined. However, from the 17th month on, as many as 85 % of both asymptomatic and symptomatic plants in the study area were infected with the virus, and a 100 % infection rate was detected in plants 18 months after the initial challenge (Fig. 2). The variation in CABMV occurrence, mainly in asymptomatic plants, is a result of the irregular distribution pattern this virus takes in passion fruit (Fischer and Rezende 2008).

### Abiotic factors in CABMV epidemiology

Abiotic factors play a role in virus epidemiology, especially in the population density of aphids in the field. Indirectly, these factors therefore affect virus spreading (Robert 1987). Here, when aphid occurrence is considered in terms of climatic conditions recorded in Pinhalzinho, the largest aphid groups were observed in spring, when mean temperature is between 20 and 24 °C (Fig. 2). These temperatures were recorded from August to late October, the period recommended for establishing passion fruit plantations in SP (Meletti 2011). During that time, rainfall did not exceed 120 mm, which is relatively low in subtropical regions. Previous research has revealed that temperatures within the 22–24 °C range and low rainfall outline a favorable scenario for most aphid species described in Brazil to form large assemblages (Carvalho et al. 2002). This condition, together with the lack of CABMV-resistant passion fruit varieties, underlines the view that, in SP, independently of the region passion fruit is grown, measures defined by phytosanitary authorities such as the use of 80-cm-tall plants to establish plantations, among others, should be followed (Meletti 2011). This recommendation is based on the need to avoid exposure of the plant to the virus during the period when young leaves are light green in color, which is less attractive to aphids (Kennedy and Stroyan 1959). This recommendation also allows the passion fruit plant leaves to fully develop a wax layer, which acts as an effective barrier against the aphids’ intracellular puncture (Meletti 2011).

In summer, when maximum daily temperatures are as high as 29 °C and mean daily rainfall is 16 mm, the number of flying aphids captured using yellow sticky traps dropped by 40 %, compared to numbers recorded in spring (Fig. 2). This may specifically be linked with temperature that, when above 25 °C, inhibits the reproduction of winged aphid forms (Minks and Harrewijn 1987). Additionally, ecological studies have revealed that these conditions do not favor the flocking of aphid species described in Brazil, since these are native to temperate zones and were introduced in the country (Ilharco 1992).

### Analysis of aphid species diversity

Fourteen aphid species of the family Aphididae were observed to fly over the passion fruit plantation surveyed (Table 1). In total, 1377 winged species were captured and identified (Table 2). Of these, 10 were polyphagous (*A. craccivora*, *A. fabae/solanella*, *A. gossypii*, *Aphis spiraecola* (Patch), *Aulacorthum solani* (Kaltenbach), *M. euphorbiae*, *M. persicae*, *Pentalonia nigronervosa* (Coquerel), *Toxoptera aurantii* (Boyer de Fonscolombe) and *T. citricidus*) and four were oligophagous (*Brevicoryne brassicae* (Linnaeus), *Nasonovia ribisnigri* (Mosley), *Pemphigus bursarius* (Linnaeus) and *U. sonchi*), which are restricted to a given botanical family (Minks and Harrewiijn 1987). Among the tribes of the family Aphididae, a 56.16 % prevalence was observed for Aphidini, followed by Macrosiphini (16.39 %) and Pemphigini (26.9 %). In Aphidini, the genus *Aphis* was the most abundant, accounting for 55.42 % of the population, in which *A. gossypii* and *A. fabae/solanella* were the most representative species observed during the survey period, mainly in winter. The Macrosiphini tribe was the most consistently represented in terms of genera (*Myzus*, *Pentalonia*, *Aulacorthum*, *Brevicoryne*, *Macrosiphum* and *Nasonovia*), of which the species *P.bursarius* was the only one of the Pemphigini tribe, though it occurred at high population numbers (Table 1). Although the number of aphid species was different, the aphid fauna composition recorded here was similar to that which observed by Kilalo et al. (2013) in passion fruit orchards in Kenya, Africa, and by Atsebeha et al. (2009) and Nault et al. (2004) in pepper crops.

**Table 1:** Taxonomic description and profile of the aphid fauna recorded in a passion fruit plantation in Pinhalzinho, São Paulo State, southeastern Brazil, between 2010 and 2011

Family	Tribus (% species)	Aphids species	Specimens (%)
Aphididae	Aphidini (56.16)	*Aphis gossypii*	(34.60)
		*Aphis fabae/solanella*	(19.00)
		*Aphis craccivora*	(1.75)
		*Toxoptera citricidus*	(0.59)
		*Toxoptera aurantii*	(0.15)
		*Aphis spiraecola*	(0.07)
	Macrosiphini (16.39)	*Aulacorthum solani*	(7.17)
		*Myzus persicae*	(4.40)
		*Macrosiphum euphorbiae*	(3.51)
		*Pentalonia nigronervosa*	(0.65)
		*Nasonovia ribisnigri*	(0.44)
		*Uroleucon sonchi*	(0.36)
		*Brevicoryne brassicae*	(0.22)
	Pemphigini (26.90)	*Pemphigus bursarius*	(26.90)

**Table 2:** Monthly and yearly winged aphid species captured between 2010 and 2011 using yellow water-pan traps in a passion fruit orchard in Pinhalzinho, São Paulo State, southeastern Brazil

Aphids species	2010								2011				
	May	Jun	Jul	Aug	Sep	Oct	Nov	Dec	Jan	Feb	Mar	Apr	T/year
*A. gossypii*	72	136	148	42	36	30	8	0	2	0	0	0	474
*P. bursarius*	204	102	22	2	3	2	2	2	4	3	3	19	368
*A. fabae/solanella*	26	37	30	35	77	32	19	0	1	0	0	3	260
*A. solani*	16	19	18	10	15	18	2	0	0	0	0	0	98
*M. persicae*	10	8	21	12	5	11	3	0	0	0	0	0	70
*M. euphorbiae*	0	4	13	12	3	7	6	0	0	1	1	1	48
*A. craccivora*	0	0	8	16	0	0	0	0	0	0	0	0	24
*P. nigronervosa*	7	0	0	1	0	0	0	0	1	0	0	0	9
*T. citricidus*	1	3	1	0	4	0	0	0	0	0	0	0	9
*N. ribisnigri*	0	0	0	0	4	1	1	0	0	0	0	0	6
*U. sonchi*	0	0	0	0	0	0	4	0	1	0	0	0	5
*B. brassicae*	0	0	0	0	0	1	2	0	0	0	0	0	3
*T. aurantii*	1	0	1	0	0	0	0	0	0	0	0	0	2
*A. spiraecola*	0	0	1	0	0	0	0	0	0	0	0	0	1
Total (T)/month	337	309	263	130	147	102	47	2	9	4	4	23	1377

It is estimated that the aphid fauna captured flying over the passion fruit plantation surveyed was formed by 12.0 % of the 116 aphid species described in Brazil (Sousa-Filho and Ilharco 1995). The population data obtained using the Shannon index (H′ = 1.788) confirmed the low aphid species diversity in the plantation surveyed. Similar results were reported by Carvalho et al. (2002), who observed the same aphid species diversity in an area cultivated with several horticultural species in Minas Gerais State (southeastern Brazil). Under subtropical conditions, this aphid fauna profile indicates the environmental alterations or degradation seen in an area used for intensive monocultures of different kinds, which reduce biodiversity and increase population levels, when compared to Atlantic Forest macro-environments, where high aphid species diversity and low population density are observed (Lazzarotto and Lazzari 1998, 2005). The results observed in the present study agree with the findings published by Dixon and Kindlmann (1990) in a study that revealed the occurrence of high aphid populations in regions where host plant diversity and richness were low, since aphids are less resourceful in finding their host plants in environments with higher plant species diversity.

### Descriptive indices used in the classification of aphid fauna

Based on the descriptive indices used to characterize the aphid fauna, which took into account the number of aphids captured in the yellow water-pan traps, it was possible to estimate that the aphid flock was formed by approximately 1,381,000 specimens/1000 m^2^ (Table 3). *A. fabae/solanella*, *A. gossypii*, *A. solani* and *P. bursarius* were commonly observed in the surveyed plantation, while *A. craccivora*, *M. euphorbiae*, *M. persicae*, *N. ribisnigri* and *T. citricidus* were intermediate species. *A. spiraecola*, *B. brassicae*, *P. nigronervosa*, *T. aurantii* and *U. sonchi* were characterized as rare (Table 4).

**Table 3:** Cumulative number of winged aphid species per square meter captured in different seasons of the year, between 2010 and 2011 using yellow water-pan traps in a passion fruit plantation in Pinhalzinho, São Paulo State, southeastern Brazil

Aphids species	Cumulative numbers of aphids m^2^ (%)			
	Spring	Summer	Autumn	Winter
*A. craccivora*	0 (0.0)	0 (0,0)	0 (0.0)	24 (3.6)
*A. fabae/solanella*	128 (43.0)	1 (6.7)	29 (8.0)	102 (14.7)
*A. gossypii*	74 (25.0)	2 (13.2)	73 (20.0)	326 (46.6)
*A. spiraecola*	0 (0.0)	0 (0.0)	0 (0.0)	3 (0.4)
*A. solani*	35 (12.0)	0 (0.0)	16 (4.3)	47 (5.7)
*B. brassicae*	3 (1.0)	0 (0.0)	0 (0.0)	0 (0.0)
*M. euphorbiae*	16 (5.6)	1 (6.7)	2 (0.5)	29 (4.2)
*M. persicae*	19 (6.4)	0 (0.0)	10 (2.8)	41 (6.1)
*N. ribisnigri*	6 (2.0)	0 (0.0)	0 (0.0)	0 (0.0)
*P. bursarius*	7 (2.4)	9 (60,0)	227 (62.0)	126 (18.0)
*P. nigronervosa*	0 (0.0)	1 (6.7)	7 (2.0)	1 (0.1)
*T. aurantii*	0 (0.0)	0 (0.0)	1 (0.2)	1 (0.1)
*T. citricidus*	4 (1,3)	0 (0.0)	1 (0.2)	4 (0.5)
*U. sonchi*	4 (1.3)	1 (6.7)	0 (0.0)	0 (0.0)
Total (m^2^)	296	15	366	704
Total (1000 m^2^)	296,000	15,000	366,000	704,000
Ranking				
1	*A. fabae/solanella*	*P. bursarius*	*P. bursarius*	*A. gossypii*
2	*A. gossypii*	*A. gossypii*	*A. gossypii*	*P. bursarius*
3	*A. solani*	*A. fabae/solanella*	*A. fabae/solanella*	*A. fabae/solanella*
4	*M. persicae*	*M. euphorbiae*	*A. solani*	*A. solani*
5	*M. euphorbiae*	*P. nigronervosa*	*M. persicae*	*M. persicae*
6	*P. bursarius*	*U. sonchi*	*T. aurantii*	*A. craccivora*
7	*N. ribisnigri*		*P. nigronervosa*	*T. citricidus*
8	*T. citricidus*			*A. spiraecola*
9	*U. sonchi*			*P. nigronervosa*
10	*B. brassicae*			*T. aurantii*

**Table 4:** General classification of winged aphid species captured using yellow water-pan traps between 2010 and 2011 in a passion fruit plantation in Pinhalzinho, São Paulo State, southeastern Brazil

Aphid species	Occurrence	Dominance	Status
*A. craccivora*	Accidental	Accessorial	Intermediate
*A. fabae/solanella*	Constant	Dominant	Common
*A. gossypii*	Constant	Dominant	Common
*A. spiraecola*	Accidental	Accidental	Rare
*A. solani*	Constant	Dominant	Common
*B. brassicae*	Accidental	Accidental	Rare
*M. euphorbiae*	Constant	Accessorial	Intermediate
*M. persicae*	Constant	Accessorial	Intermediate
*N. ribisnigri*	Accessorial	Accidental	Intermediate
*P. bursarius*	Constant	Dominant	Common
*P. nigronervosa*	Accidental	Accidental	Rare
*T. aurantii*	Accidental	Accidental	Rare
*T. citricidus*	Accessorial	Accidental	Intermediate
*U. sonchi*	Accidental	Accidental	Rare

Analyzing the occurrence and dominance indices, *A. gossypii* was one of the four most representative species that fly over passion fruit plantations, together with the polyphagous species *A. fabae/solanella, A. solani* and *M. persicae* that, independently of the season, were classified as common species (Table 4). As for oligophagous species, the prevalence of *P. bursarius* was recorded in the fall and in the winter, and it was also characterized as a common species (Tables 3, 4).

In spring, the period recommended for the establishment of passion fruit plantations in SP, the most abundant species were *A. fabae/solanella* and *A. gossypii* (Table 3) that, together with *A. solani* and *P. bursarius*, was classified as common species, that is, they account for more than 50 % of the species collected (Table 4). During this season, polyphagous (*M. persicae* and *M. euphorbiae*) and oligophagous (*N. ribisnigri* and *T. citricidus*) aphids were also abundant and ranked as intermediate species (Tables 3, 4). Then, *B. brassicae* and *U. sonchi*, which were accidental species, were categorized as rare species (Table 4).

Green-tile traps are more selective than yellow water-plan traps, and have been used to study the dynamics of aphids involved in the transmission of several viruses transmitted in a non-persistent manner (De Banno 1991; Irwin and Kampmeier 1989). Here, the result obtained using these traps allowed estimating the number of aphids landing on the passion fruit plantation surveyed at 61,000 specimens/1000 m^2^, which corresponds to 4.7 % of the aphid fauna that flew over the plantation. In turn, diversity obtained using this trap was represented by only eight species (*A. fabae/solanella*, *A. gossypii*, *A. solani*, *M. euphorbiae*, *M. persicae*, *P. bursarius*, *T. citricidus* and *U. sonchi*) (Table 5). It was also observed that landings were more frequent in the winter. The general classification indices revealed that only *U. sonchi* was rare, while the other species were intermediate and predominantly polyphagous. None of the aphid species captured using the Irwin trap were classified as common (Table 6). As a consequence of the ecological panorama of the aphid fauna recorded in the winter, the number of CABMV-infected passion fruit plants increased from 40 % to 60 % in spring (2011).

**Table 5:** Cumulative number of winged aphid species per square meter captured in different seasons of the year, between 2010 and 2011 using Irwin green tile traps in a passion fruit plantation in Pinhalzinho, São Paulo State, southeastern Brazil

Aphid species	Cumulative numbers of aphids m^2^ (%)			
	Spring	Summer	Autumn	Winter
*A. fabae/solanella*	2 (66.6)	0 (0.0)	2 (33.3)	14 (28.2)
*A. gossypii*	1 (33.4)	0 (0.0)	2 (33.3)	11 (22.6)
*A. solani*	0 (0.0)	0 (0.0)	0 (0.0)	13 (26.7)
*M. euphorbiae*	0 (0.0)	0 (0.0)	0 (0.0)	5 (10.3)
*M. persicae*	0 (0.0)	0 (0.0)	0 (0.0)	3 (6.1)
*P. bursarius*	0 (0.0)	0 (0.0)	1 (16.7)	3 (6.1)
*T. citricidus*	0 (0.0)	2 (66.6)	1 (16.7)	0 (0.0)
*U. sonchi*	0 (0.0)	1 (33.4)	0 (0.0)	0 (0.0)
Total (m^2^)	3	3	6	49
Total (1000 m^2^)	3000	3000	6000	49,000
Ranking				
1	*A. fabae/solanella*	*T. citricidus*	*A. gossypii*	*A. fabae/solanella*
2	*A. gossypii*	*U. sonchi*	*A. fabae/solanella*	*A. solani*
3			*P. bursarius*	*A. gossypii*
4			*T. citricidus*	*M. euphorbiae*
5				*M. persicae*

**Table 6:** General classification of winged aphid species captured using green tile traps between 2010 and 2011 in a passion fruit plantation in Pinhalzinho, São Paulo State, southeastern Brazil

Aphid species	Occurrence	Dominance	Status
*A. fabae/solanella*	Accidental	Dominant	Intermediate
*A. gossypii*	Accidental	Dominant	Intermediate
*A. solani*	Accidental	Dominant	Intermediate
*M. euphorbiae*	Accidental	Dominant	Intermediate
*M. persicae*	Accidental	Accessorial	Intermediate
*P. bursarius*	Accidental	Dominant	Intermediate
*T. citricidus*	Accidental	Accessorial	Intermediate
*U. sonchi*	Accidental	Accidental	Rare

### The aphid fauna associated with CABMV dissemination

Considering that aphid interaction with host plants and the reproduction strategy of one aphid species are influenced by the region where the population is established (Eastop 1977), and that parthenogenesis is common in tropical and subtropical regions, where polyphagous species diversity is higher (Minks and Harrewiijn 1987), the results obtained reveal that, independently of the season, the aphid fauna in Pinhalzinho may potentially become a CABMV transmission agent in passion fruit plantations. From the epidemiological standpoint, this condition favors the consolidation of this virus in these environments, mainly due to non-persistent transmission. This scenario is favored by the fact that the monoculture of fruit, vegetable, and ornamental species is increasingly adopted in eastern SP, apart from the conservation of large areas covered with both invader and native species to the region. These regional characteristics play an important ecological role, and directly influence abundance and seasonal occurrence of aphid species (Wolda 1978).

**Fig. 3:**
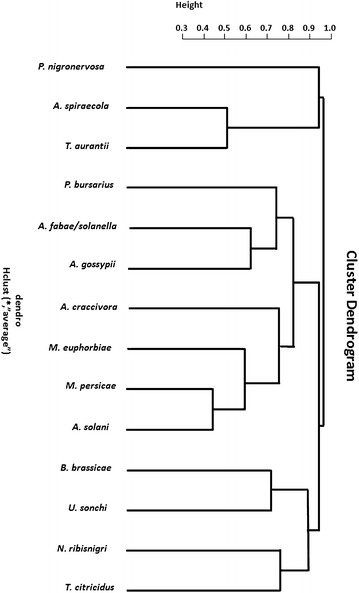
Dendrogram showing two distinctive clusters of aphid species constructed using the Jaccard index in the R software. The similarity pattern established for the analysis was based on the diversity parameters and the total number of aphid species captured using yellow water pan traps between 2010 and 2011 in a passion fruit plantation in Pinhalzinho, eastern São Paulo State, southeastern Brazil

The Jaccard index and the descriptive indices revealed the trend of aphids to cluster as two distinct communities, formed by different species. One of these clusters was characterized by the high diversity of polyphagous aphid species categorized as common or intermediate and that may become potential CABMV vectors, independently of season. In turn, the second cluster was formed by two polyphagous species (*A. spiraecola* and *T. aurantii)* and one oligophagous species (*P. nigronervosa*), which were classified as rare and therefore less relevant in CABMV dissemination (Fig. 3).

However, in spite of the favorable conditions to spread CABMV, the virus was detected in the passion fruit plantation by PTA-ELISA only from the 13th month on, after introduction in the field. Therefore, it becomes clear that, despite the high polyphagous aphid species numbers in the surveyed plantation throughout the year, the occurrence of CABMV is relatively low in eastern SP, which helps the translocation of passion fruit plantations to other areas. However, despite the record of frequent flocks of aphids as of the beginning of monitoring, it is likely that the delayed establishment of the virus in the orchard was a consequence of the low CABMV inoculum pressure, which in turn results from the recent introduction of passion fruit culture in eastern SP.

Due to the economic importance of passion fruit and to the lack of information on the role aphid species as virus vectors, especially in large tropical and subtropical regions, as in Brazil, the results obtained confirm the importance of bioecological inventories that afford a more in-depth understanding of the relationship between aphid communities and the environment they live in, shedding more light on the consequences of this relationship as far as disease transmission is concerned (Lewisnohn et al. 2005).

During the monitoring period, despite the low aphid fauna diversity, a relatively high biological divergence in aphid species was observed, based on the predominance of polyphagous species, followed by oligophagous ones. This had a direct effect on the ecological relationships in the colonization or non-colonization of host plants in the field. Therefore, flocks flying over the passion fruit orchard were also affected. However, the tropical and subtropical conditions in Brazil, associated with the absence of alternative host plant species, may favor the colonization of passion fruit plants by the polyphagous species *A. gossypii* and *T. aurantii* (Sousa-Filho and Ilharco 1995; Di Piero et al. 2006). This was not observed during the present survey. Yet, in the sampling efforts carried out in the natural vegetation growing in the orchard, *S. oleraceus* was seen to host *U. sonchi*, while *S. americanum* were hosts to *A. fabae/solanella* (data not shown). For Moran et al. (1999) and Peccoud et al. (2010), *Uroleucon* species are oligophagous and specifically colonize representatives of the family Asteracea. This behavior was observed in the passion fruit orchard surveyed, which explains the low numbers of *U. sonchi* flocks and the ranking of the species as rare. On the other hand, *A. fabae*, which was classified as polyphagous, was recorded as larger flocks, and was ranked as common. The prevalence of *A. fabae* colonies in *S. americanum* individuals also allowed concluding that these species’ flocks were actually formed by the subspecies *solanella*, since currently *A. fabae* has six subspecies, depending on specificity to host plants: *cirsiiacanthoidis*, *eryngii*, *evonymi*, *fabae*, *mordvilkoi* and *solanella* (http://www.faunaeur.org)*. Aphis (Aphis) fabae* Scopoli 1763. Retrieved 2014/11/24). In light of the 14 aphid species with distinct biological and ecological characteristics, it was not feasible to obtain and maintain pure colonies of all aphid species that could be assessed for their efficiency as CABMV vectors. In this sense, in order to estimate the epidemiological role of *U. sonchi* and *A. fabae/solanella* in CABMV spreading, considering the distinct biological characteristics, transmission assays were carried out only for these two species.

Transmission tests using the CABMV isolate obtained in the plantation surveyed revealed that *U. sonchi* is rare, and that it did not transmit CABMV. This finding confirms the minor importance of this aphid species in CABMV transmission in eastern SP. However, *A. fabae/solanella*, which often was reported colonizing *S. americanum* throughout the monitoring period and was categorized as a common species, exhibited a 40 % efficiency to transmit CABMV (Table 7). Atiri et al. (1986) used *Aphis* species in CABMV transmission assays in leguminous plants and observed that *A. craccivora*, *A. gossypii* and *A. spiraecola* have mean transmission efficiency of 50 %. Conversely, in a study on the epidemiological aspects of CABMV in passion fruit plants, Di Piero et al. (2006) found that transmission efficiency of *A. gossypii* may be as high as 75–100 %, depending on the number of individuals in the flock. Similar results were obtained here, for the interaction of *A. fabae*/*solanella* with CABMV and passion fruit plants. Therefore, these results underscore the view that *Aphis* are indeed effective CABMV vectors, mainly when compared with other polyphagous, oligophagous and monophagous aphid species (Atiri et al. 1986). In a survey carried out in the State of Minas Gerais, Brazil, *S. oleraceus* was reported to be colonized by a large number of polyphagous aphid species, of which the most representative were *M. euphorbiae* and *M. persicae* (Carvalho et al. 2002). These aphids have also been described to be efficient CABMV vectors (Inoue et al. 1995). Nevertheless, *A. fabae/solanella*, which was often detected colonizing *S. americanum* (data not shown) and that ranked as common species in the plantation surveyed, was likewise an efficient CABMV vector, as revealed by the transmission tests. The role of CABMV vector played by the genus *Aphis* had been reported by Di Piero et al. (2006), in a study that also revealed that passion fruit plants may be colonized mainly by *A*. *gossypii*, under trophic stress conditions. It was found that the aphids that colonize these plants may migrate to other species, including passion fruit. All in all, in the absence of an ideal host plant, *A. fabae*/*solanella* may colonize passion fruit, acting as CABMV inoculum reservoirs, which makes it an important agent of virus spreading. In terms of seasonal abundance of aphids relative to passion fruit orchards, *Aphis* spp. are the most important in the epidemiology of CABMV.

**Table 7:** Efficiency of the transmission of the *Cowpea aphid*-*borne mosaic virus* (CABMV) to passion fruit plants using *Aphis fabae/solanella* and *Uroleucon sonchi*

Aphid species	Symptoms/absorbance (A_405_)		
	Rep 1	Rep 2	Rep 3
*A. fabae/solanella* (n)			
(1)	+/1.125	−/0.336	−/0.166
(2)	−/0.287	+/0.852	−/0.201
(3)	+/0.980	+/1.157	+/0.844
(4)	+/1.345	−/0.507	−/0.196
(5)	−/0.489	−/0.880	−/0.196
*U. sonchi* (n)			
(1)	−/0.293	−/0.140	−/0.238
(2)	−/0.263	−/0.225	−/0.213
(3)	−/0.155	−/0.165	−/0.191
(4)	−/0.147	−/0.249	−/0.194
(5)	−/0.129	−/0.340	−/0.163
Positive control	0.890		
Negative control	0.249		

In Brazil, due to the lack of CABMV-tolerant or resistant passion fruit varieties, the translocation of passion fruit plantations to regions where the virus is not yet consistently prevalent, independently of the aphid populations present there, is one of the management practices recommended. In this scenario, the combination of the preventive control of aphid populations before large flocks are formed, the management of Asteraceae and Solanaceae invader plants, the use of tall plants, and the reallocation of plantations are measures that may stall CABMV spread in eastern SP. The implementation of such initiatives may allow keeping the same plantation for as long as 2 years, an advantage that nevertheless has not proved itself to be viable in areas in SP where CABMV is endemic, where producers are required to systematically renew plantations once a year (Meletti 2011).

However, the practical results of this recommendation for passion fruit intensive production settings are incipient, since, according to Kranz (1974), most surveys carried out in economically important cultures overlook the true concept of epidemiology: studies are essentially based on the disease and its causal agent, and fail to address the need for a more comprehensive understanding of the interactions between pathogens, the environment, host populations, and vectors.

## Conclusion

We have observed that the low abundance and diversity of aphid species did not interfere negatively in the CABMV epidemiology. The genus *Aphis*, particularly *Aphis fabae/solanella* and *A. gossypii*, was crucial in the spread of CABMV in passion fruit orchards in the eastern State of São Paulo. However, the translocation of passion fruit plantations to new regions, where the virus is not yet consistently prevalent, independently of the aphid populations, is one of the management practices recommended for the control of the passionfruit woodiness disease. *A. fabae*/*solanella*, classified here as a common species was an efficient vector of CABMV, while *U. sonchi*, classified as a rare species was not able to transmit the virus. Maintaining of CABMV in the monitored area was related to the presence of weeds, that were colonized by aphids, and responsible for the maintenance of flights on passion fruit orchard. The passion fruit orchards and weeds are important in the epidemiology of CABMV, mainly during the spring and autumn, when transient aphid vectors are searching for new hosts. The fact that aphid species trapped in autumn differ from those present in spring, probably does not account for such differences because high numbers of potential vectors of CABMV land on the crop during both seasons.
